# Epidemiological characteristics of *Candida *species colonizing oral and rectal sites of Jordanian infants

**DOI:** 10.1186/1471-2431-11-79

**Published:** 2011-09-09

**Authors:** Shireen Y Issa, Eman F Badran, Kamal F Akl, Asem A Shehabi

**Affiliations:** 1 Department of Pathology-Microbiology, Faculty of Medicine, University of Jordan, 11942, Amman, Jordan; 2 Pediatrics/Division Neonatology, Jordan University Hospital, 11942, Amman, Jordan

**Keywords:** *Candida *colonization, virulence, genotypes, antifungal susceptibility

## Abstract

**Background:**

There is evidence that *Candida *colonization contributes to increasing invasion of candidiasis in hospitalized neonates. Few studies investigated the epidemiology and risk factors of *Candida *colonization among hospitalized and non-hospitalized infants. This prospective study investigated the major epidemiological characteristics of *Candida *species colonizing oral and rectal sites of Jordanian infants.

**Methods:**

Infants aged one year or less who were examined at the pediatrics outpatient clinic or hospitalized at the Jordan University Hospital, Amman, Jordan, were included in this study. Culture swabs were collected from oral and rectal sites and inoculated on Sabouraud dextrose agar. All *Candida *isolates were confirmed by the Remel RapID yeast plus system, and further investigated for specific virulence factors and antifungal susceptibility MIC using E-test. Genotyping of *C. albicans *isolates was determined using random amplified polymorphic DNA (RAPD) analysis method.

**Results:**

A total of 61/492 (12.4%) infants were colonized with *Candida *species by either their oral/rectal sites or both. Rectal colonization was significantly more detected than oral colonization (64.6% verses 35.4%), particularly among hospitalized infants aged more than one month. The pattern and rates of colonization were as follows: *C. albicans *was the commonest species isolated from both sites and accounted for 67.1% of all isolates, followed by *C.kefyr *(11.4%), each *C. tropicalis *and *C. glabrata *(8.9%) and *C. parapsilosis *(3.8%).

A various rates of *Candida *isolates proved to secrete putative virulence factors *in vitro*; asparatyl proteinase, phospholipase and hemolysin. *C. albicans *were associated significantly (P < 0.05) with these enzymes than other *Candida *species. All *Candida *isolates were susceptible to amphotericin B and caspofungin, whereas 97% of *Candida *species isolates were susceptible to fluconazole using E-test.

The genetic similarity of 53 *C. albicans *isolates as demonstrated by dendrogram revealed the presence of 29 genotypes, and of these one genotype accounted for 22% of the isolates.

**Conclusion:**

This study presents important epidemiological features of *Candida *colonization of Jordanian infants.

## Background

*Candida *colonization of infants is a risk factor for developing candidiasis, especially in neonatal intensive care unit (NICU) [1-4]. During the past decade colonization and candidaemia with *non-albicans Candida *species has risen dramatically with high rates of carriage in hospitalized infants including neonates admitted to NICU [2,5,6].

It is generally observed that most infant candidiasis is thought to be endogenously acquired through prior colonization of different parts of the body, while other studies reported that certain outbreaks of *Candida *infection were caused by nosocomial infection in neonatal intensive care units [7-10].

The potential pathogenesis of *Candida *species appears to depend on many immunological and environmental host factors and strain virulence factors, including hyphae and biofilm formation, drug resistance and the production of extracellular hydrolytic enzymes [11-15].

Phenotyping and genotyping of *Candida *isolates are important features can be used to investigate the common genotypes and possible route of transmission and infection within hospitals and community, especially molecular typing of *Candida *isolates is highly useful tool in detection source of nosocomial infections [16-19]. This study was carried out to investigate the major epidemiological characteristics of *Candida *colonizing hospitalized and non-hospitalized Jordanian infants.

## Methods

This prospective study was conducted at the Jordan University Hospital over a period of 10-month; from March 2008 to December 2008. The study has been approved by the high graduate committees of the Faculty of Medicine, and ethics committee of Jordan Hospital University and Faculty of Graduate studies/University of Jordan, Amman, Jordan. Verbal consent was obtained from all mothers of infants after explaining the purpose of the study.

### Study participants

A total of 492 infants (aged one year or less) were included in this study over a period of 10-month (2008-2009) as follows: Group 1; 265 neonates admitted to neonatal intensive care unit (NICU). Group 2; 37 infants admitted to pediatric ward (PW) due to urinary tract or kidney problems. Group 3; 190 infants examined in pediatric outpatients clinic. For each patient important demographic characteristics were recorded on special study form, and all hospitalized neonates and infants were observed for developing candidaemia.

### Specimen collection

Pre-wetted cotton swabs with sterile saline were used to collect culture specimens from oral and rectal sites of each infant. For neonates in the NICU; oral and rectal swabs were collected within 24 hr of birth, day seven and after every one week until the baby discharged or died. Fresh oral and rectal specimens were handled and inoculated directly on Sabouraud dextrose agar plates (SDA, Oxoid, Ltd, Basingstoke, UK) which supplemented with chloramphenicol (0.05 g/l) and incubated at 37°C for 24-48 hrs.

### Mycological investigations

All growth of yeast-like colonies was subsequently identified by subculture 2-3 representative colonies on a CHROMagar Candida medium (Oxoid, Ltd, Basingstoke, UK) and incubated at 37°C for 24-48 hr. Candida growth was identified by detection of various color characteristics on CHROMagar Candida plates [20]. All Candida species isolates were confirmed by the Remel RapID yeast plus system (Remel Inc, Lenexa, KS). Reference standard strains of *C. albicans *(ATCC 90028), *C. glabrata *(ATCC 22553) and *C. parapsilosis *(ATCC 22019) were subcultured on the same medium as controls.

Detection of extracellular production of aspartyl proteinases was made for all *Candida *isolates by demonstration and measurement of the clear zone of proteolysis around *Candida *colony growth in bovine serum albumin agar [20]. Production of extracellular phospholipaes activity was estimated by growing *Candida *on egg-yolk agar and observing the precipitation zone around the *Candida *colony growth [12]. Beta-hemolysin production was evaluated using a fresh human blood agar plate and after incubation for 48 hr [20]. Reference strains of *C. albicans *(ATCC 10231) served as positive control for proteinase and phospholipaes assays and *C. albicans *(ATCC 90028) served as positive control for haemolysin assay.

### Antifungal susceptibility test

Etests were performed according to the manufacturer's instructions (AB Biodisk, Sweden). The antifungal agents used were amphotericin B, fluconazole and caspofungin. A quality control *C. albicans *strain (ATCC 90028) was included.

### Genotyping

Determination of *Candida *genotype was performed using random amplified polymorphic DNA (RAPD) analysis method and PCR amplification with three random oligonucleotides single primers T3B primer 5'-d(AGG TCG CGG GTT CGA ATCC) 3' [21]. RSD 10 primer 5'-d(CCG CAG CCA)-3' and RSD 12 primer 5'-d(GGT CCG TGT TTC AAG ACG)-3' [22].

### Statistical analysis

All Data analysis were performed using the computerized statistical program Statistical Package of Social Science program (SPSS, version 16, USA) and was used to determine the P values and investigated phylogenetic tree (dendrogram) showing the genetic relatedness among the isolates which was constructed based on genetic similarities. In all statistical tests, the differences were considered to be statistically significant if p-value (< 0.05).

## Results and Discussion

Demographic characteristics of 492 investigated infants with total positive and negative *Candida *species cultures are shown in Table 1. This study showed that *Candida *colonization was recorded in 12.4% of all infants, and rectal site was significantly more colonized than oral site (64.6% vs 35.4%; P < 0.05). *Candida *colonization was significantly more prevalent among hospitalized infants aged ≥ 30 days than neonates admitted to NICU (15.3% vs 10.6%). Despite this fact only one case of candidaemia has been detected to be associated with *Candida *colonization in a hospitalized neonate over the 10-month study period (Table 1). The study also demonstrated that age, gender, the duration of hospitalization, previous antibiotic treatment of infants were not a significant risk factor associated with *Candida *colonization (Table 1). *C. albicans *was the commonest species (67.1%) isolated from both oral and rectal sites of infants, whereas other *non-albicans Candida *species accounted for one third of isolates (Table 2). It is difficult to correlate the results of this study with most other studies which have been investigated mainly the relationship between risk factors of *Candida *colonization and developing of *Candida *infections in neonates hospitalized in NICU [1,3-5,9]. However, a study in Greece has found that *Candida *species colonization was detected in 12.1% of neonates during a 12-month period, and *C. albicans *was isolated from 42% of colonized neonates. In addition, candidemias were diagnosed more in colonized neonates (6.9%) as compared with 0.76% of noncolonized neonates (P = 0.002)[3]. A recent study from Brazil reported that 19% of the neonates were colonized by *Candida *species which were divided equally between *C. albicans *(50%) and non-*albicans Candida *(50%) [1]. The increased colonization of non-*albicans Candida species *as well as their cause of candidaemia in neonates and adult Jordanian patients has been shown to be similar to other studies from various countries [1,2,6,4,7,23].

**Table 1:** Demographic Characteristics of 492 investigated infant with Positive *Candida *colonization from oral/rectal or both specimens

Variables	*Candida *Colonized infants No. (%)	P-value
**Age by group**		
0 - 28 days (neonates)	28/265(10.6)	0.135
29 days - 1 year (infants)	33/227(15.3)	
Total	61/492(12.4)	
**Gender**		
Male	35/256 (13.7)	0.764
Female	26/236(11.0)	
**Patients by group**		
NICU	18/265(6.8)*	0.001
Pediatric ward	12/37(32.4)	
Outpatients	31/190(16.3)	
**Hospital Stay**		
1 - 7 days	13/138(8.6)	0.373
8 - 30 days	13/116(10.1)	
> 30 days	4/18(18.2)	
**Antibiotic treatment**		
Yes	30/285(10.5)	0.139
No	31/207(15.0)	

* One hospitalized neonate was his oral and rectal sites colonized

with *C. albicans *has developed Candidemia

**Table 2:** Distribution of *Candida *species isolates colonizing oral and rectal sites of 61 infant patients*

Type of *Candida*	Oral colonization No. (%)	Rectal colonization No. (%)	Total colonization No. (%)
***C.albicans***	21 (75.1)	32 (62.7)	53 (67.1)
***C.kefyr***	1 (3.6)	8(15.7)	9 (11.4)
***C.tropicalis***	2 (7.1)	5 (9.8)	7 (8.9)
***C.glabrata***	2 (7.1)	5 (9.8)	7 (8.9)
***C.parapsilosis***	2 (7.1)	1 (2.0)	3 (3.8)
**Total**	28 (35.4)	51 (64.6)**	79 (100)

* Includes 18 infant patients have both oral and rectal colonization at the same time.

** Significant (P < 0.05)

All *Candida *isolates in this study were 100% susceptible to amphotericin B and caspofungin, while susceptibility to fluconazole was observed only in 5/7 *C. glabrata *isolates (Table 3). These results are similar to some extent to a previous study published from Jordan [23].

**Table 3:** Antifungal susceptibility results of the 79 oral and rectal *Candida *isolates

Candida species (no. of isolates)	% susceptible(MIC range/mg/L)		
	Amphotericin B	Fluconazole	Caspofungein
***C. albicans*** **(53)**	100 (0.002 - 1.5)	100 2-16))	100 (0.064-1)
***C. glabrata*** **(7)**	100 (0.002 - 0.75)	**7**1.6* (4-48)	100 0.25-75))
***C. kefyr*** **(9)**	100 0.5 - 1.5)**)**	100 1.5 - 3))	100 (0.125-0.75)
***C. tropicalis*** **(7)**	100 (0.125 - 75)	100 (2**-**4)	100 (0.25-1)
***C. parapsilosis*** **(3)**	100 (0.38-1.0)	100 (1-6)	100 (0.5-75)

* 2/7 of *C.glabrata *isolates were resistant to fluconazol

The present study has detected a significant production of putative virulence enzymes of phospholipase, protease in most *C. albicans *isolates from oral and rectal specimens, compared to production of these enzymes among non-albicans *Candida *species (Table 4). The expression of hemolysin activity was also significant among the majority of *C. albicans, C. tropicals, C. glabrata *compared to other *Candida *isolates. However, no significant relationship has been detected between *Candida isolates *from oral or rectal specimens and exertion of these enzymes or in relation to their antifungal susceptibility. Many studies have suggested that hemolytic activity and hydrolytic enzymes are putative virulence factor contributing to *Candida *colonization and hematogeneous infection [12,14,15,20,24]. A recent study has shown that the increased pathogenicity of *Candida *drug-resistant strains for systemic infection was associated with a number of biochemical and physiological changes, including cellular alterations in cell wall polysaccharides, rapid and extensive hypha and biofilm formation [25].

**Table 4:** Distribution of proteinase, phospholipase and hemolysin among *Candida *species isolates

Enzyme	*Candida species*	No. of isolates	Positive isolates No. (%)	Negative isolates No. (%)	P value
**Proteinase**	*C. albicans*	53	49 (92.5)	4 (7.5)	0.004
	*C. kefyr*	9	6 (66.7)	3 (33.3)	
	*C. tropicalis*	7	4 (57.1)	3 (42.9)	
	*C. glabrata*	7	4 (57.1)	3 (42.9)	
	*C. parapsilosis*	3	1 (33.3)	2 (66.7)	
**Total**		79	64 (81.0)	15 (19.0)	
**Phospholipae**	*C. albicans*	53	48 (90.6)	5 (9.4)	0.0001
	*C. kefyr*	9	7 (77.8)	2 (22.2)	
	*C. tropicalis*	7	3 (42.9)	4 (57.1)	
	*C. glabrata*	7	2 (28.6)	5 (71.4)	
	*C. parapsilosis*	3	1 (33.3)	2 (66.7)	
**Total**		79	61 (77.2)	18 (22.8)	
**Hemolysin**	*C. albicans*	53	45 (84.9)	8 (15.1)	0.0012
	*C. kefyr*	9	3 (33.3)	6 (66.7)	
	*C. tropicalis*	7	7 (100)	0	
	*C. glabrata*	7	6(85.7)	1(14.3)	
	*C. parapsilosis*	3	1 (33.3)	2 (66.7)	
**Total**		79	62 (78.5)	17 (21.5)	

**Figure 1:**
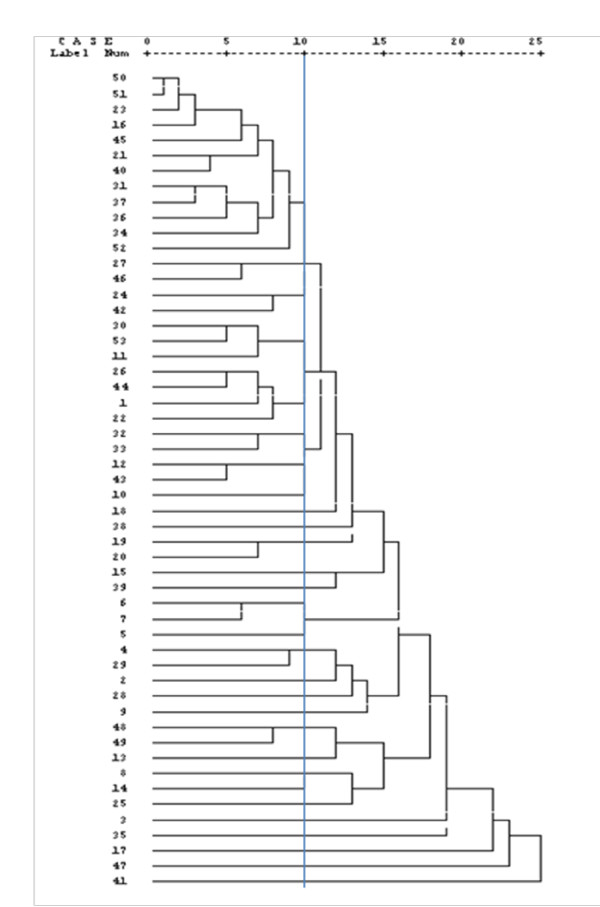
**Dendrogram of 53 *C. albicans *isolates**.

The results of RAPD patterns of *C. albicans *isolates as demonstrated in constructed dendrogram suggest that at least one genotype was prevalent (22.6%) among all colonized infants either hospitalized or not (Figure 1,2 &3). It has been proved tha RAPD is a very useful method for evaluating and comparing the genetic profiles of *C. albicans *clones [22].

**Figure 2:**
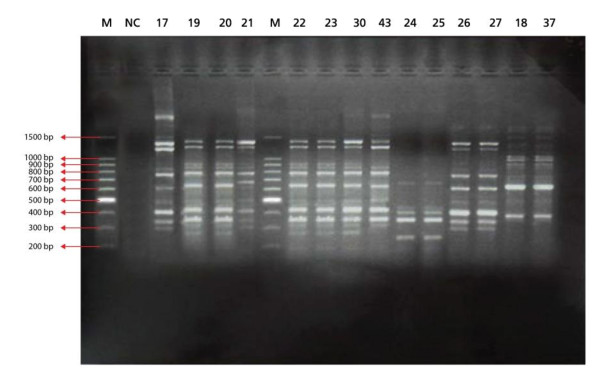
**RAPD-PCR patterns generated by *C. albicans *isolates using T3B primer, M, 100 bp PCR DNA marker; Lane 1, negative PCR blank; Numbered lanes show patterns of different representative *C. albicans *isolates**.

**Figure 3:**
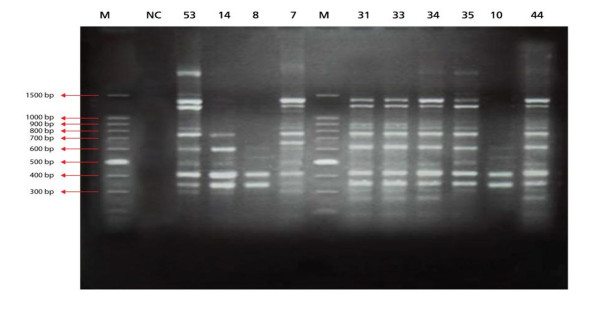
**RAPD-PCR patterns generated by *C. albicans *isolates using RSD 10 primer, M, 100 bp PCR DNA marker; Lane NC, negative PCR blank**. Numbered Lanes show patterns of different representative *C. albicans *isolates.

## Conclusion

This study contributes to increase our understanding of the epidemiology of *Candida *colonization in neonates and infants whether hospitalized or not.

## Competing interests

The authors declare that they have no competing interests.

## Authors' contributions

AS and EM have written the conception and design of the study and drafted the final manuscript for publication. EM and KA have supervised all clinical investigations and data collection of the examined infants. SI and AS were responsible for performing all laboratory tests, data analysis and statistical analysis. All authors read and approved the final manuscript.

## Pre-publication history

The pre-publication history for this paper can be accessed here:

http://www.biomedcentral.com/1471-2431/11/79/prepub
